# Investigating the impact of lumping heterogenous conduct problems: aggression and rule-breaking rely on distinct spontaneous brain activity

**DOI:** 10.1007/s00787-024-02557-w

**Published:** 2024-08-14

**Authors:** Jules Roger Dugré, Stéphane Potvin

**Affiliations:** 1 School of Psychology and Centre for Human Brain Health, University of Birmingham, Birmingham, B15 2TT United Kingdom; 2 Department of Psychiatry and Addiction, Faculty of medicine, University of Montreal, Montreal, Canada; 3 Centre de recherche de l’Institut Universitaire en Santé Mentale de Montréal, 7331, Hochelaga, Montreal, H1N 3V2 Canada

**Keywords:** Resting-state, Conduct problems, Aggression, Rule-breaking, Neuroimaging

## Abstract

Accumulating evidence suggests that aggression and rule-breaking may have distinct origins. However, grouping these heterogeneous behaviors into a single dimension labelled Conduct Problems (CP) has become the norm rather than the exception. Yet, the neurobiological features that differentiate aggression and rule-breaking remain largely unexplored. Using a large sample of children and adolescents (*n* = 1360, 6–18 years old), we examined the common and specific brain activity between CP, aggression, and rule-breaking behaviors. Analyses were conducted using fMRI resting-state data from a 10-minute session to explore the correlations between low frequency fluctuations and both broad and fine-grained CP dimensions. The broad CP dimension was associated with deficits in the precentral gyrus, superior temporal gyrus, and tempo-parietal junction. However, only the superior temporal gyrus was shared between aggression and rule-breaking. Activity of the precentral gyrus was mainly associated with rule-breaking, and the temporo-parietal cortex with aggression. More importantly, voxel-wise analyses on fine-grained dimensions revealed additional specific effects that were initially obscured when using a broad CP dimension. Finally, we showed that the findings specific to aggression and rule-breaking may be related to distinct brain networks and mental functions, especially ventral attention/sensorimotor processes and default mode network/social cognitions, respectively. The current study highlights that aggression and rule-breaking may be related to distinct local and distributed neurobiological markers. Overall, using fine-grained dimensions may provide a clearer picture of the role of neurobiological correlates in CP and their invariance across measurement levels. We advocate for adopting a more thorough examination of the lumping/splitting effect across neuroimaging studies on CP.

**Supplementary Information:**

The online version contains supplementary material available at 10.1007/s00787-024-02557-w.

## Introduction

In the past few years, a growing interest for dimensional approaches has been observed in psychopathology research [43]. For a multitude of psychopathologies, analyses favor dimensional latent structure over binary categories [34]. While the dimensional structure of psychopathologies has been well described [41], the impact of employing a broad level of measurement on our understanding of psychopathologies has yet to be uncovered. For example, conduct problems (CP) are generally defined as a serious disregard for and violation of others’ rights and societal norms, including aggressive behaviors towards individuals or animals, theft/robbery, and property destruction [9]. Despite enduring evidence from research and clinical practice that advocates for distinguishing aggression from rule-breaking syndromes [4–6, 35, 58]^1^^,^^2^^,^^3^, lumping these behaviors under a single taxon (i.e., Conduct Disorder [CD]) and/or dimension (i.e., CP) [7–9, 52] has now become the norm rather than the exception [63]. One of the main arguments for lumping these heterogeneous behaviors is their frequent co-occurrence (approximately 30–63.9% of children, [10, 68] and their moderately-to-strongly correlation (*r* ≈ 0.40-0.60, [16]. Even if researchers found that both dimensions may load onto a broader latent factor labelled “*antisocial behaviors*” or “*conduct problems*” [42], aggression and rule-breaking appears to be distinct [12, 62]. For instance, by lumping heterogeneous behaviors (e.g., CP), researchers assume that their findings would consistently correlate with the more fine-grained level of measurements (e.g., aggression, rule-breaking) [54]. Here, we attempted to describe how measuring aggression and rule-breaking separately may provide a clearer picture of our understanding of the neurobiological mechanisms of CP.

### Clinical phenotypes: prioritizing a more fine-grained measurement level

Intriguingly, a growing body of research underscores the substantial differences between aggression and rule-breaking in terms of their etiological influences, developmental trajectories, and risk factors. Indeed, meta-analytic findings from twin studies suggest that genetic influences primarily account for antisocial behaviors and CP (≈ 0.49), followed by non-shared environmental influences (≈ 0.37), and shared environmental (≈ 0.14) [31, 49, 57, 59]. However, aggression and rule-breaking appear to only share 38.4% of their genetic underpinnings [16]. More precisely, most of the variance in aggression is explained by genetic influences (up to 65%), whereas environmental influences appear to be greater for rule-breaking compared to aggression (18–22% versus 5–6%, respectively) [16, 59]. These findings may partially be explained by the cumulative evidence showing that aggression may be more strongly associated to temperamental features (e.g., emotional lability), whereas rule-breaking may rather be associated with environmental and socio-normative factors during adolescence including stimulation-seeking, substance use, and affiliation with delinquent peers [18, 25, 39, 44, 53, 55, 64]. Additionally, both behaviors are characterized by distinct developmental trajectories. For instance, aggression typically manifest in early childhood and decline throughout adolescence, while rule-breaking behaviors are rare in childhood but show a sharp increase in mid-adolescence [19]. Despite these distinctions, both behaviors likely arise from complex gene-environment interplay [17] involving numerous multi-systemic risk and protective factors such as family (e.g., parental involvement, low physical punishment), school (e.g., school achievement, support), and neighborhood factors (e.g., cohesion) [17, 45]. While both aggression and rule-breaking behaviors frequently co-occur, these findings underscore the importance of studying their shared and specific aetiologies through various lenses including neurobiology.

Recent meta-analytic evidence from functional neuroimaging studies indicates that individuals exhibiting antisocial behaviors may show widespread deficits in brain activity during threat detection, cognitive control, social cognition, and punishment processing [29]. However, the substantial variations in the selection of fMRI tasks limit the generalizability of the findings. Hence, over the last decades, an increasing popularity has been reported for measuring the brain activity and functional connectivity at rest [66]. For example, several groups have shown that during resting-state, children and adolescents with a CD demonstrate deficits in spontaneous brain activity of various subcortical (i.e., amygdala, thalamus), parietal (i.e., postcentral gyrus, posterior superior temporal sulcus, inferior parietal lobule), frontal (i.e., orbitofrontal cortex, precentral gyrus), occipital (i.e., lingual, cuneus), temporal and cerebellar structures [46, 67, 71]. These partially align with recent meta-analytic findings indicating that individuals exhibiting antisocial behaviors show variations in functional connectivity of regions the default-mode network (DMN), ventral attention, visual, and somatomotor networks [24]. Importantly, both fMRI measures complement our understanding of brain functioning underlying CP. While spontaneous brain activity (amplitude of brain signal) provides insight into potential local deficits in brain resources, functional connectivity (correlation between time-series of brain regions) highlights potential disruptions in information propagation across brain regions. However, linking results on brain activity and functional connectivity remains highly challenging. To address this limitation, some have recently demonstrated that the heterogeneous peak coordinates found across fMRI studies on youths with CD (38 studies) may map onto a common network [28] which involved regions of the subcortex, DMN, visual, and somatomotor networks. Intriguingly, these findings partially align with those reported by a previous meta-analysis of resting-state studies among antisocial populations [24], suggesting that the regional deficits may be crucial for understanding the disrupted functional connectivity underlying CD. However, none of the previous fMRI studies investigated whether brain activity correlates of CD might be differently influenced by aggression and rule-breaking, even though both dimensions are associated with distinct etiological influences, risk factors and developmental trajectories. Nevertheless, some have reported that the functional connectivity correlates between aggression and rule-breaking appear seemingly identical when using the total scores on Child Behavior Checklist [21]. These shared neural features mainly include functional connectivity of the DMN, dorsal attention, and limbic networks [21]. In contrast, Ibrahim and colleagues [38] showed that aggression was associated with functional connectivity among regions of the somatomotor network, visual network, and salience network, as well as between regions of the visual network and those of the salience and frontoparietal networks. Using partial least square, others have reported that items related to rule-breaking may be mainly characterized by functional connectivity among regions of the visual network, salience network, and DMN [65]. Taken together, these findings suggest that aggression and rule-breaking may share several neural features among regions of the DMN (e.g., posterior superior temporal sulcus, inferior parietal lobule), visual (lingual, cuneus), and limbic (e.g., orbitofrontal cortex) networks, but also show distinct effects among regions of the somatomotor network (pre & postcentral gyri).

Despite important differences in the etiology and developmental trajectory of aggression and rule-breaking behaviors, neurobiological research investigating their shared and distinct correlates are lacking. Identifying their neurobiological mechanisms may provide additional insights about the heterogeneity among youths exhibiting CP. Therefore, we sought to investigate the effect of studying the neurobiological correlates of a broad CP dimension and whether it could potentially obscure meaningful associations when using more fine-grained dimensions, namely aggression and rule-breaking. The study aimed to achieve two main objectives: (A) examine whether the neural correlates found for a broad CP dimension are linked to both (or specific) aggression and rule-breaking, and (B) to pinpoint neural markers that may be specific to each of the behavioral manifestations. More specifically, we examined the associations between the broad CP dimension and spontaneous brain activity (i.e., fractional amplitude low-frequency fluctuation [f/ALFF]). Then, we conducted additional analyses on composite score of rule-breaking and aggression. Given that deficits in spontaneous brain activity may be informative of potential disruption in functional connectivity [28], we conducted additional analyses to identify what brain areas are functionally connected to regional deficits underpinning each dimension. Based on the limited number of published studies, we nevertheless hypothesized that the broad CP would be associated with deficits in brain activity of regions of the DMN. Aggression and rule-breaking would be similarly associated with deficits in the DMN, but aggression may be more strongly associated with variations in brain regions of the somatosensory network [38, 65].

## Methods

### Participants

Data from 2200 participants were obtained from the Healthy Brain Network (HBN), an ongoing initiative in New York area (USA) that aims to investigate heterogeneity and impairment in developmental psychopathology (5–21 years old) [2]. The HBN adopted a community-referred recruitment model in which advertisements was provided to community members, educators, and parents. Exclusion criteria were impairments that prevented full participation in the study (e.g., serious neurological disorders, hearing or visual impairments), neurodegenerative disorder, acute encephalopathy, acute intoxication, and serious psychiatric disorders (recent diagnosis of schizophrenia and/or manic episode). Additionall information is provided elsewhere [2]. From the 2200 participants included in the Data Release 7.0, 1583 participants contained available functional neuroimaging data. Written assent was obtained from participants younger than 18 years old, and written consent was obtained from their legal guardians. Written informed consent was obtained from participants aged 18 or older prior to enrolling in the study. The original HBN study was approved by the Chesapeake Institutional Review Board (now Advarra Inc., see https://www.advarra.com/). The current study was approved by the local ethics committee of Centre intégré universitaire de santé et de services sociaux (CIUSSS) de l’Est-de-l’Île-de-Montréal.

#### Modelling the latent structure of conduct problems

To model the latent structure of CP, the full sample was first split in two subsamples based on the availability of neuroimaging data. The subsample without available functional neuroimaging data (*n* = 617) was first used as a training sample for Exploratory Factor Analysis (EFA) with a promax rotation, followed by a Confirmatory Factor Analysis (CFA) on the subsample with available neuroimaging data (*n* = 1583). Both samples did not differ on any of the demographic or clinical variables (Table S1) except age (*p* < 0.001). However, means, standard deviations and ranges of non-imaging (M = 10.2, SD = 3.07, range = 6.01–17.83) and imaging (M = 10.86, SD = 3.09, range 6.00-17.95) samples were highly similar. More importantly, both samples showed no significant differences in severity of CBCL-CP items (17 items, Table S2).

Promax rotation was chosen over orthogonal rotation given the well-established correlation strength between AGG and RB. These analyses were conducted using the DSM-Oriented CP scale (17 items) as assessed by the Parent-Report Child Behavior Checklist (CP-CBCL 6/18, [1]. Parents rated each item using a 3-point scale (0 = not true to 2 = very true) (α = 0.84-0.87). Items showing a tetrachoric correlation ≥ 0.65 were excluded before running the EFA due to redundancy and multicollinearity. We removed the item showing the greatest average inter-item correlation of the item-pair. We assessed the EFA adequacy using Bartlett’s test of sphericity and Kaiser-Meyer-Olkin criterion. The most optimal number of factors was extracted by finding the maximum curvature of the scree plot. Only items with loadings > 0.4 were retained to keep items that differentiate aggression from rule-breaking the most. These analyses were conducted using SPSS version 29 and python version 3.10.

Using the left-out sample, a CFA was conducted on the remaining set of items. More precisely, we tested (1) a unidimensional CP model and (2) a correlated factors model (aggression, rule-breaking). Model fit was assessed using the root mean square error of approximation (RMSEA), the comparative fit index (CFI) and the Tucker-Lewis index (TLI). We also assessed the composite reliability of the broad and fine-grained dimensions. These analyses were carried out using MPLUS version 6.

#### Neuroimaging acquisition parameters

MRI acquisition took place at three different sites: mobile 1.5T Siemens Avanto in Staten Island, 3T Siemens Tim Trio at Rutgers University Brain Imaging Center (RUBIC), and 3T Siemens Prisma at the CitiGroup Cornell Brain Imaging Center (CBIC) (acquisition protocols and parameters can be found in Table S3, in [2] as well as http://fcon_1000.projects.nitrc.org/indi/cmi_healthy_brain_network/). Data at the CBIC were obtained using the same data acquisition protocol implemented at RUBIC which included two resting-state sessions lasting 5 min each during which participants viewed a fixation cross located at the center of the computer screen. These two runs were concatenated. Data for the Siemens Avanto were acquired in a single run lasting 10 min.

#### Preprocessing of resting-state fMRI data

The preprocessing steps are described elsewhere [26] and can be found in supplementary method. Preprocessed images were manually checked for each of the 1583 participants. We found pre-processing issues due to the poor quality of images in 108 participants, which resulted in the software unable to adequately detect & segment volumes into tissue classes (i.e., grey matter, white matter, and cerebrospinal fluid). In addition, 59 adolescents exhibited high movements (exceeding 3 mm) leaving a remaining sample of 1416 participants. Finally, given that the CBCL measures children and adolescent psychopathologies (< 18 years old), 56 adult subjects were excluded, leaving a final sample size of 1360 adolescents (Table 1).

**Table 1:** Characteristics of the final sample for subsequent neuroimaging analyses

Characteristics	Final Sample (*n* = 1360)
Age	10.79 (3.16)
Sex (Males, %)	789 (62.1%)
Recruitment Site	
Staten Island	295 (21.7%)
RUBIC	612 (45%)
CBIC	453 (33.3%)
Ethnicity	
White	651 (47.9%)
Black	224 (16.5%)
Hispanic	154 (11.3%)
Bi/Multiracial	238 (17.5%)
Others	92 (6.8%)
Percentage of Valid Scans (mean %, s.d.)	86.6% (17.3%)
Mean motion (mean mm, s.d.)	0.34 (0.46)

### Statistical analyses

#### Amplitude of low frequency oscillations

Amplitude Low frequency fluctuation (ALFF) aims to assess the strength of spontaneous neural activity within the low frequency band [70]. ALFF is calculated as the root mean square of BOLD signal at each individual voxel after band-pass filtering [69]. Although it shows moderate to high test-retest reliability [73], ALFF is sensitive to physiological noise. Therefore, we decided to use fractional ALFF (fALFF) which is conducted by taking the power of low frequency range divided by the total power of entire frequency range [72]. Linear regression was first conducted to examine the main effect of CP-Composite score on fALFF values across the whole brain, while adjusting for age, sex, sites, percentage of valid scans, and mean motion. We then tested whether the resulting CP-related fALFF findings were also correlated with more fine-grained behavioral subtypes. Secondly, given that a more fine-grained measurement level may lead to additional findings that are specific to behavioral subtypes, we ran additional linear regression using aggression and rule-breaking composite scores on fALFF across the whole brain, while adjusting for covariates. Statistical significance was determined via a threshold of *p* < 0.001 uncorrected with 20 voxels. Findings that meet a more stringent correction were also reported (*p* < 0.001 with a family-wise (FWE) correction of *p* < 0.05 at a cluster-level). Results of the ALFF measure are reported in Table S6.

#### Normative functional connectivity map

Recently, we have used normative samples to identify how peak coordinates of brain activity underpinning CD may be linked to a common network [56, 61]. In the current study, we adopted similar approach and used resting-state data of healthy individuals to investigate the whole-brain connectivity profile of fALFF results for each dimension. Data from 687 healthy children and adolescents from the Autism Brain Imaging Data Exchange datasets (ABIDE) I [22] and II [23] were used. These participants had no self-reported history of autism or and their absence of past or current psychiatric or neurological disorders was determined via unstructured detailed interview. Resting-state functional connectivity data were preprocessed using identical preprocessing steps as the HBN sample (see Supplementary Methods). Preprocessed images were manually checked. We applied a stringent threshold regarding motion and excluded 89 subjects (i.e., average framewise displacement > 0.20 and/or percentage of remaining scans after scrubbing less than 75%). This left a remaining sample of 598 participants (mean age = 11.87, s.d.= 2.77; 71.6% boys; mean framewise displacement = 0.12 mm, s.d.=0.038; mean percentage valid volumes = 97%, s.d. = 4.33%). More information about the samples can be found in Table S4) as well as elsewhere (https://fcon_1000.projects.nitrc.org/indi/abide/).

First, we modelled the fALFF regions as seeds (4 mm sphere) and performed seed-to-voxels analyses on ABIDE datasets to generate a whole-brain functional connectivity network for each dimension. Average time course of voxels within seeds for each dimension were correlated with the time-course of every voxel in the brain for each of the 598 healthy participants. Correlation coefficients were converted to normally distributed z-values using Fisher’s z-transformation. A one-sample t-test was then conducted across the functional connectivity map of the 598 subjects to create the functional connectivity profile for each dimension. For each dimension-specific map, we examined the main contribution of 8 intrinsic functional connectivity networks including Schaefer-400 parcels 7 Networks [60] and a subcortical network [32]. Effect sizes (Cohen’s *d*) were computed by extracting the mean intensity of voxels within each intrinsic network and comparing it to the mean intensity of voxels outside the given network. We additionally investigated the potential mental functions underpinning the dimension-specific functional connectivity map by computing spatial correlation (adjusted for spatial autocorrelation) with 13 mental functions derived from a data-driven analysis on 1,347 task-based fMRI meta-analyses [27].

## Results

### Latent structure of conduct problems

In the EFA, none of the 17 items showed correlation | ≥ 0.65 | (max = 0.56, min=-0.04), suggesting low redundancy & multicollinearity. Bartlett’s test of sphericity (χ2 = 2640.18, df = 136, *p* < 0.001) and the Kaiser-Meyer-Olkin criterion (KMO = 0.87) suggested suitability for factor analysis. Scree plot suggested a two-factor solution as the most optimal number of components (Figure S1; Table S5).

**Fig. 1:**
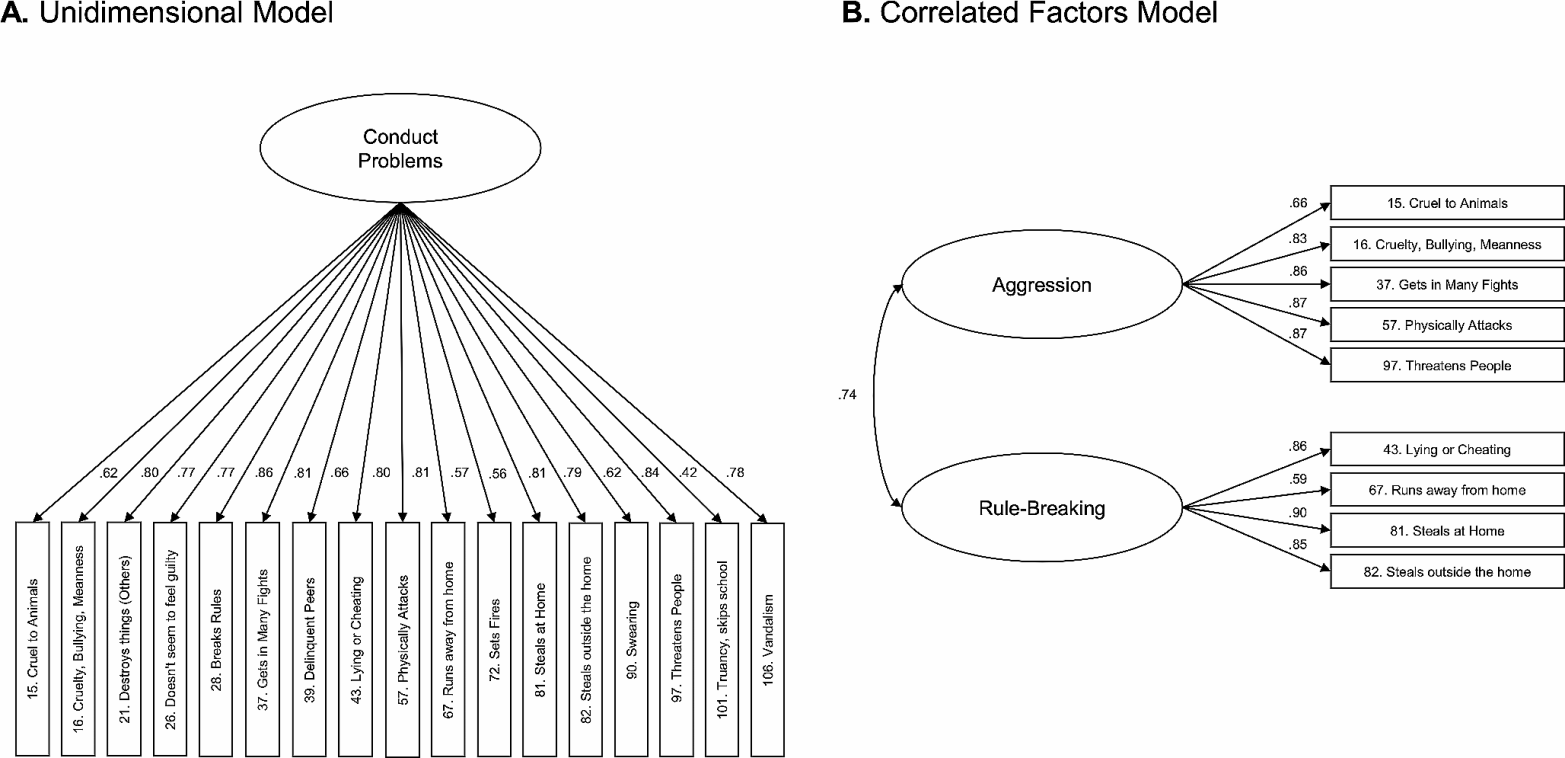
Confirmatory Factor Analysis for Unidimensional and Correlated Factors Models Across Conduct Problems in Adolescents

Overall, the CFA revealed an excellent fit for both correlated factor model (RMSEA, value = 0.035, 90% confidence interval [CI] = 0.025-0.046; CFI, value = 0.991; TLI, value = 0.988) and the unidimensional CP model on the original DSM-Oriented 17 items (RMSEA = 0.046, CI: 0.042-0.051; CFI = 0.971, TLI = 0.967). The three derived dimensions also showed excellent reliability: aggression (CR = 0.912) and rule-breaking (CR = 0.881) and CP (CR = 0.95) (Fig. 1).

### Fractional amplitude low frequency fluctuation (fALFF)

#### Neural correlates of unidimensional conduct problems

**Fig. 2:**
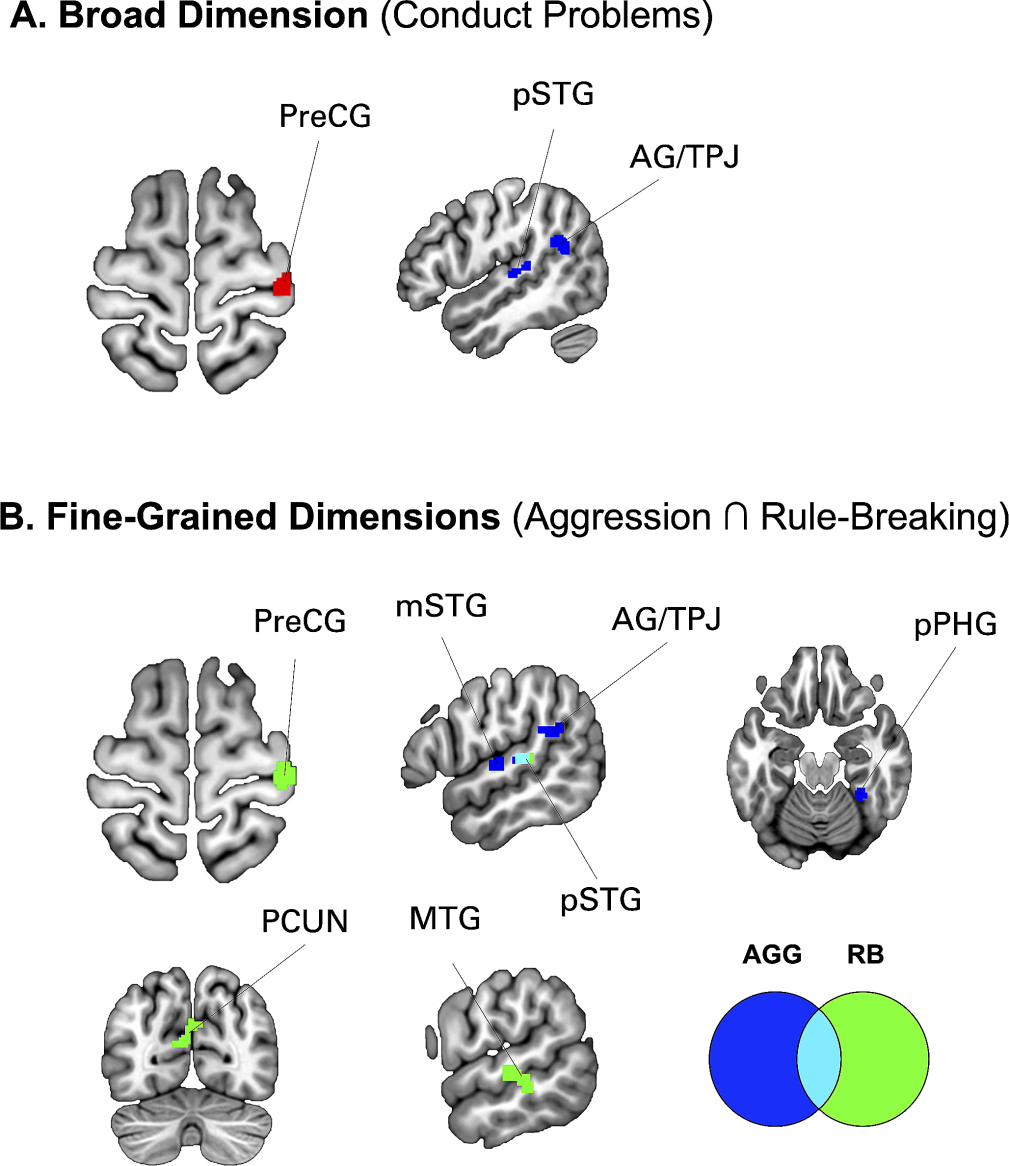
Associations between Fractional Amplitude of Low-Frequency Fluctuations and Conduct Problems. **(A)** Associations with unidimensional composite score. Red = Positive correlation; Blue = Negative correlation. PreCG = Precentral Gyrus; pSTG = Posterior Superior Temporal Gyrus; AG/TPJ = Angular Gyrus/Temporo-Parietal Junction. **(B)** Associations with fine-grained dimension (behavioral subtypes). Blue = Aggression Composite Score; Green = Rule-Breaking Composite Score; Cyan = Overlap between Aggression and Rule-Breaking. mSTG = mid-Superior Temporal Gyrus; pPHG = posterior Parahippocampal Gyrus; PCUN = Precuneus; MTG = Middle Temporal Gyrus

Modelling CP as a single broad dimension revealed positive association with fALFF in the right precentral gyrus (PreCG) (*r* = 0.10, *p* < 0.001), and negative associations with fALFF in the right posterior superior temporal gyrus (pSTG) (*r*=-0.13 < 0.001) and angular gyrus/temporoparietal junction (AG/TPJ)(*r*=-0.14, *p* < 0.001) (see Fig. 2; Table 2). These findings were also replicated with controlling for the effect of internalizing traits (Table S7). Also similar findings were found when examining ALFF (Figure S2, Table S7). We then extracted fALFF values for these cluster and conduct partial correlation with aggression and rule-breaking composite scores while adjusting for covariates. Both aggression and rule-breaking were associated with fALFF in the PreCG (r_AGG_=0.091, *p* < 0.001; r_RB_=0.12, *p* < 0.001), pSTG (r_AGG_=-0.14, *p* < 0.001; r_RB_=-0.12, *p* < 0.001) and TPJ (r_AGG_=-0.12, *p* < 0.001; r_RB_=-0.11, *p* < 0.001).

**Table 2:** Fractional Amplitude Low Frequency Fluctuations (fALFF) findings

Brain Regions (Composite Score)	Direction (+/-)	MNI Coordinates			t-value	cluster size
		x	y	z		
**SHARED EFFECT** (CP ∩ AGG ∩ RB)						
**Superior Temporal Gyrus (posterior)**						
Conduct Problems	-	56	-26	8	-4.48	42
Aggression	-	56	-26	8	-4.76	32
Rule-Breaking	-	56	-26	8	-4.52	34
**AGGRESSION EFFECT** (AGG ∩ CP; AGG-specific)						
**Temporo-Parietal Junction (Inferior Parietal Lobule)**						
Conduct Problems	-	56	-26	8	-4.52	34
Aggression	-	48	-38	22	-3.54	22
**Mid Superior Temporal Gyrus**						
Aggression	-	60	-10	4	-4.69	37
**Posterior Parahippocampal Gyrus**						
Aggression	-	32	-42	-18	-4.10	23
**Primary Motor Cortex**						
Aggression	+	-10	-14	74	4.29	22
**RULE-BREAKING EFFECT** (RB ∩ CP; RB specific)						
**Precentral Gyrus**						
Conduct Problems	+	38	-24	66	3.59	22
Rule-Breaking	+	40	-24	66	3.98	46
**Middle Temporal Gyrus**						
Rule-Breaking	-	-58	-30	-2	-3.76	30
**Precuneus**						
Rule-Breaking	-	0	-70	26	-3.53	32

*Note* Findings are thresholded at *p* < 0.001, 20 voxels. Models are adjusted for effect of age, sex, sites, percentage of valid scans and mean motion. MNI = Montreal Neurological Institute

### Neural correlates of fine-grained behavioral phenotypes

We then sought to investigate whether using fine-grained behavioral phenotypes would result in additional regional effects that would have been blurred by combining heterogenous constructs under the broad CP dimension.

Voxelwise linear regression using Aggression-Composite score replicated the CP-related fALFF findings in the pSTG (*r*=-0.13, *p* < 0.001) and TPJ (*r*=-0.12, *p* < 0.001) (see Table 2; Fig. 2). Analyses revealed additional findings including positive association with fALFF of the primary motor cortex (M1)(*r* = 0.11, *p* < 0.001) and negative association with fALFF of the mid-STG (*r*=-0.13, *p* < 0.001) and posterior Parahippocampal Gyrus (PHG) (*r*=-0.12, *p* < 0.001) (see Table 1; Fig. 2). These were replicated after controlling for the effect of internalizing traits (Table S6). ALFF analyses also showed deficits in frontal areas and secondary somatosensory cortex (Figure S2, Table S7).

Linear regression using Rule-Breaking Composite score replicated the CP-related fALFF findings in the pSTG and the PreCG. Analyses additionally revealed negative association with fALFF in the middle temporal gyrus (MTG) (*r*=-0.13, *p* < 0.001) and precuneus (PCUN) (*r*=-0.12, *p* < 0.001) (see Table 2; Fig. 2). These were replicated after controlling for the effect of internalizing traits (Table S6). ALFF analyses showed additional deficits in visual and cerebellar regions (Figure S2, Table S7).

### Aggression and rule-breaking behaviors are linked to distinct overarching brain networks

We then explored whether aggression and rule-breaking may differ at a regional level (fALFF deficits) but but still be associated to a common overarching functional connectivity network. Consequently, for each dimension, we identified the functional connectivity profile of fALFF clusters across 598 healthy adolescents (see Table 2; Fig. 2).

**Fig. 3:**
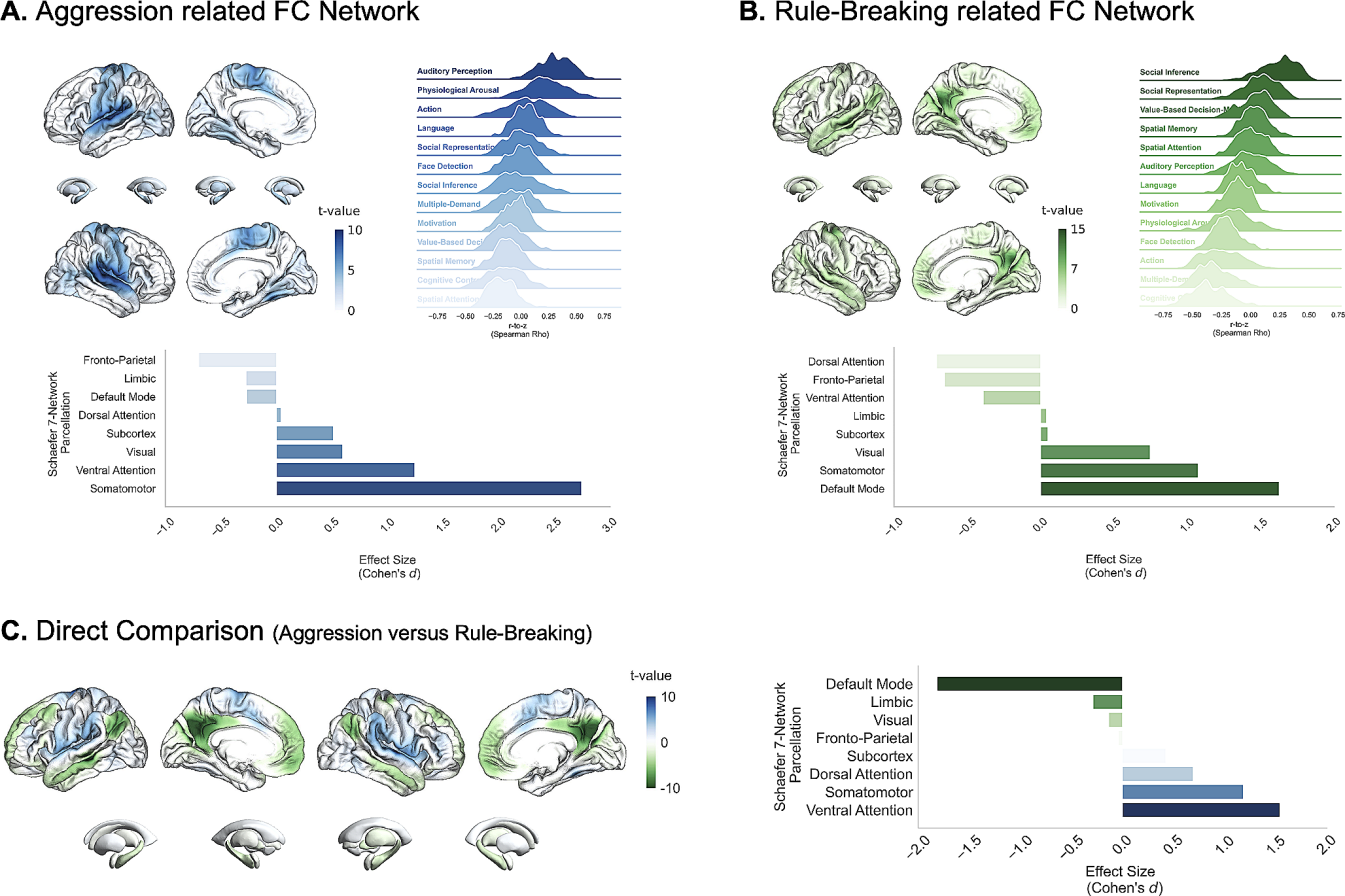
Functional Connectivity profiles of the fALFF deficits for **(A)** Aggression, **(B)** Rule-Breaking behaviors, and **(C)** Direct contrast between aggression and rule-breaking behaviors. Normative Functional Connectivity Maps were calculated from the fractional amplitude of low frequency fluctuations (fALFF) findings for both fine-grained dimensions. Ridge plots represent the distribution of correlation between functional connectivity maps and mental functions (Dugré et Potvin, 2023) among the 598 healthy adolescents of the ABIDE datasets. Bar Graphs represent the effect sizes (Cohen’s d) corresponding to the importance of each intrinsic connectivity network in the aggression and rule-breaking maps

First, we observed that the fALFF deficits associated with the broad dimension of CP was primarily driven by functional connectivity of the somatomotor (*d* = 2.72), ventral attention (*d* = 1.22) networks (Figure S3, Table S8 for complete results). Regarding aggression, similar effect was found for the somatomotor (*d* = 2.74) and ventral attention (*d* = 1.24) networks but additionally show moderate importance of visual (*d* = 0.59) and subcortical (*d* = 0.51) networks. In contrast, fALFF deficits associated with rule-breaking behaviors were rather functionally connected to the DMN (*d* = 1.63), somatosensory (*d* = 1.07), and visual (*d* = 0.75) networks.

To better interpret these findings, we investigated the spatial association between these functional connectivity maps and 13 maps of distinct mental functions. We observed that the functional connectivity network underpinning the broad unidimensional dimension of CP was primarily associated with by Auditory Perception (Fisher z-transformation of spearman rho z’=0.57, pFDR = 0.0006), Physiological Arousal (z’=0.39, pFDR = 0.0006), and Action (z’=0.13, pFDR = 0.03) (Figure S3, Table S9 for complete results). Regarding aggression related functional connectivity map, similar effect was observed for Auditory Perception (z’=0.51, pFDR = 0.0009), Physiological Arousal (z’=0.35, pFDR = 0.0009), and Action (z’=0.29, pFDR = 0.0009). However, Rule-Breaking functional connectivity was only statistically associated social inference processes (e.g., false beliefs) (z’=0.40, pFDR = 0.002).

Direct contrast between aggression and rule-breaking related functional connectivity maps revealed stronger importance of the ventral attention and somatomotor for the former, and the DMN for the latter (see Fig. 3, Table S8). Moreover, spatial associations with mental functions revealed stronger association with somatosensory processes (i.e., Auditory Perception, Physiological Arousal, Action) for the former, and socio-affective processes (i.e., Social Inference, Value-Based decision-making, Social Representation) for the latter (see Table S9).

## Discussion

The current study aimed to examine the impact of lumping conduct problems in our understanding of the neurobiological correlates of behavioral subtypes. Despite strong evidence that aggression and rule-breaking may be distinct, researchers still consider these behaviors as a unidimensional construct. Here, we demonstrated that using a broad level of measurement (CP) may blur meaningful associations that were observed at a more fine-grained level. First, we found that CP was associated with spontaneous brain activity in the pSTG, PreCG, and AG/TPJ, also found in ALFF analyses. Investigating the brain correlates of fine-grained dimensions replicated the brain results found for CP, but also revealed additional important findings. Indeed, aggression composite score was associated with amplitude of the midSTG, pPHG, and M1, while rule-breaking yielded significant effects in middle temporal gyrus and precuneus. While we expected some degree of overlap, these findings underscore the importance of employing a more fine-grained approach to disentangle the neurobiological correlates of CP and call for cautious interpretation when modeling CP as a single dimension.

During resting-state, children and adolescents with a CD appear to show regional variations in subcortical (i.e., amygdala, thalamus), parietal (i.e., PoCG, pSTS/IPL), frontal (i.e., OFC, PreCG), occipital (i.e., lingual, cuneus), temporal and cerebellar structures [46, 67, 71]. In our study, we replicated some of these findings by showing that the broad CP dimension was significantly correlated with the amplitude of the PreCG, pSTG, and AG/IPL. More importantly, we found that only the fALFF of the pSTG significantly correlated with the broad and fine-grained behavioral phenotypes at a voxel-level (*p* < 0.001 uncorrected, 20 voxels). Intriguingly, this exact location, commonly referred as the primary auditory cortex [11], is frequently found across antisocial population. Indeed, brain activity of the pSTG has been found in children with disruptive behavior disorders [40, 47] and adults with psychopathy [3, 33, 50] and correlate with severity of CD [30]. Moreover, morphological brain abnormalities of the pSTG are also reported across studies on reactive aggression [74] and in children with CD [36] and particularly in girls [51] and women with prior CD [13, 14]. One possibility is that variations in this sensory area may confers a greater vulnerability to attribute hostile intentions to other’s speech. Indeed, a recent study found that activity of the pSTG correlated with attributing hostile intention to laughter which was further linked to the severity of aggression [48]. Furthermore, we showed that some of the findings from the literature on CD might be better explained by a specific behavioral subtype. Indeed, we observed that CP-related fALFF variations in the PreCG was replicated in rule-breaking (but not aggression), and the AG/TPJ in aggression (but not rule-breaking). For example, changes in the AG/IPL [46] may be specifically related to aggression, whereas variations in the PreCG, MTG, and cerebellar [67] may be rather associated with rule-breaking. Overall, these findings suggest that using a more fine-grained measurement level may potentially provide a clearer picture of the neurobiological correlates of CP and their invariance across measurement level. We encourage researchers to undertake additional analytic steps to investigate whether their findings on broad CP measures might be shared or specific to particular behavioral dimensions.

Antisocial behaviors are likely to be characterized by widespread activity in various brain networks including the DMN, somatomotor, visual and ventral and dorsal attention [24]. Darby and colleagues [20] recently found that heterogeneous brain lesions characterizing criminal behaviors may be related to a common overarching network of neural alterations in adults. This network mainly overlapped with the DMN, and morality and value-based decision-making meta-analytic maps. In our study, despite that aggression and rule-breaking may be related to changes in activity of distinct brain regions, they could potentially be functionally connected to a common brain network. Using resting-state connectivity data from a large independent sample of healthy youths, we demonstrated that the main fALFF effects associated with severity of CP may reflect changes in functional connectivity of the somatomotor and DMN networks. Indeed, fALFF regions of both aggression and rule-breaking also showed functional connectivity to the somatomotor network, suggesting a shared effect. However, we further observed that the fALFF findings of rule-breaking were specifically associated with functional connectivity to the DMN, while aggression may be mainly driven by ventral attention networks. These effects may partially explain the different mental processes underpinning both behaviors. Indeed, this align with prior work highlighting the importance of prospection/planning in general delinquency [15, 53], and salience/ventral attention and somatomotor networks in childhood aggression [37]. Our findings indicate that fine-grained dimensions may be related to shared yet distinct underlying neural mechanisms and warrant further examination.

### Limitations

Despite the strengths of our study aiming to examine the effects of lumping CP into a single dimension, some limitations need to be acknowledged. For instance, the sample used in this study contains a relatively wide age range spanning from childhood to late adolescence. Here, we controlled for age given the potential confounding effects on our results. However, it is possible that some results might be specific to developmental stages (e.g., childhood vs. adolescence). Second, neuroimaging data was collected in 3 different sites (two with identical scanning parameter) that may have altered results. Third, the HBN adopted a community-referred recruitment model, and careful interpretations of our results should be made when comparing with population-based cohorts. Fourth, despite the usefulness of identifying distinct functional connectivity profile between aggression and rule-breaking, future studies should aim to replicate the fALFF differences between both dimensions and to test whether dimensions are significantly associated with distinct functional connectivity networks.

## Conclusion

For an identical severity score of CP, one participant may report a high score on aggression but low on rule-breaking while the other may report the opposite. Consequently, using a broad CP dimension which comprise heterogeneous constructs may reduce the strength of relationship with neurobiological markers and even blur out some findings that were specific to aggression or rule-breaking. The choice of measurement level (i.e., using a broad over fine-grained dimensions) is of utmost importance to our understanding of the neurobiological markers of children with CD which need to be justified throughout literature. To prevent obscuring critical findings, one suggested approach would be to begin investigating the commonality and differences in findings between the fine-grained phenotypes and subsequently test whether they are comparable or not with at a broader measurement level. Consequently, researchers should consider this important methodological issue before choosing to use a broad CP dimension and should, at the very least, examine whether their findings are shared or specific between dimensions.

## Electronic supplementary material

Below is the link to the electronic supplementary material.

Supplementary Material 1

## Data Availability

Data is available here https://fcon_1000.projects.nitrc.org/indi/cmi_healthy_brain_network/ and https://fcon_1000.projects.nitrc.org/indi/abide/.

## Footnotes

1 In 1968, the DSM-II referred to children/adolescents with Conduct Problems as: 308.3) Runaway reaction; 308.4) Unsocialized Aggressive Reaction, and 308.5) Group Delinquent Reaction.

2 In 1980, the DSM-III referred to children/adolescents with Conduct Disorder as: 312.00) Undersocialized – Aggressive; 312.10) Undersocialized – Nonaggressive; 312.23) Socialized – Aggressive; 312.21) Socialized – Nonaggressive.

3 In 1987, the DSM-III-TR referred to children/adolescents with Conduct Disorder as 312.00) Solitary Aggressive, 312.20) Group Type, 312.90) Undifferentiated.
