# Supplementary material for: Investigating the impact of lumping heterogenous conduct problems: aggression and rule-breaking rely on distinct spontaneous brain activity
Source: Eur Child Adolesc Psychiatry. 2024 Aug 14;34(3):1207–19. doi: 10.1007/s00787-024-02557-w (PMC11909054; doi:10.1007/s00787-024-02557-w)
Supplement: Supplementary file 1 — Supplementary Material 1 [file 787_2024_2557_MOESM1_ESM.docx]

– SUPPLEMENTARY MATERIAL –

**Investigating the Impact of Lumping Heterogenous Conduct Problems:**

**Aggression and Rule-Breaking rely on distinct Spontaneous Brain Activity**

**Abbreviated title:** Should we Lump or Split Conduct Problems in Neuroimaging?

Jules R. Dugré, PhD ^1^, & Stéphane Potvin, PhD ^2,3^

^1^ School of Psychology and Centre for Human Brain Health, University of Birmingham,

Birmingham

^2^ Research Center of the Institut Universitaire en Santé Mentale de Montréal, Montreal, Canada

^3^ Department of Psychiatry and Addictology, Faculty of medicine, University of Montreal, Montreal, Canada

**Corresponding authors**

Jules Roger Dugré, PhD

Centre for Human Brain Health, University of Birmingham, School of Psychology, Birmingham B15 2TT

Email: j.dugre@bham.ac.uk.

&

Stéphane Potvin, PhD;

Centre de recherche de l’Institut Universitaire en Santé Mentale de Montréal; 7331 Hochelaga; Montreal, Canada; H1N 3V2;

Email: [stephane.potvin@umontreal.ca](mailto:stephane.potvin@umontreal.ca)

**Table of contents**

[**Supplementary Methods** 3](#_Toc171611664)

[**Supplementary Tables** 4](#_Toc171611665)

[**Table S1.** Difference in Demographic and Clinical variables between Imaging and Non-Imaging samples (HBN sample) 4](#_Toc171611666)

[**Table S2.** Difference in Conduct Problems between Imaging and Non-Imaging samples 5](#_Toc171611667)

[**Table S3.** MRI Scan Parameters of the Healthy Brain Network dataset 6](#_Toc171611668)

[**Table S4.** Information about the Autism Brain Imaging Data Exchange datasets of Healthy Subjects (n=598) 7](#_Toc171611669)

[**Table S5**. Results of the exploratory factor analysis (pattern matrix) 8](#_Toc171611670)

[**Table S6.** Analyses on fALFF correlates after considering Internalizing Traits as a potential confounder 9](#_Toc171611671)

[**Table S7.** Findings from the Amplitude Low Frequency Fluctuations (ALFF) analyses. 10](#_Toc171611672)

[**Supplementary Figures** 14](#_Toc171611673)

[**Figure S1.** Scree plot revealed a maximum curvature at 2 Factors 14](#_Toc171611674)

[**Figure S2.** Findings from the Amplitude Low Frequency Fluctuations (ALFF) 15](#_Toc171611675)

[**Figure S3.** Normative Functional Connectivity Map derived from the regional deficits fALFF for Conduct Problems. 16](#_Toc171611676)

[**Supplementary Results** 17](#_Toc171611677)

# **Supplementary Methods**

Preprocessing of Resting-state fMRI data

Functional images were realigned, corrected for motion artifacts with the Artifact Detection Tool [3](ART, setting a threshold of 0.9 mm subject ART’s composite motion and a global signal threshold of Z = 5) with the implemented in CONN Toolbox [4], bandpass filtered (0.01 Hz < f < 0.10 Hz) and co-registered to the corresponding anatomical image. The anatomical images were segmented (into grey matter, white matter, and cerebrospinal fluid) and normalized to the Montreal Neurological Institute (MNI) stereotaxic space. Functional images were then normalized based on structural data, spatially smoothed with a 6 mm full-width-at-half-maximum (FWHM) 3D isotropic Gaussian kernel and resampled to 2 mm^3^ voxels. For the preprocessing, the anatomical component-based noise correction method (aCompCor strategy, [1]), was employed to remove confounding effects from the BOLD time series, such as the physiological noise originating from the white matter and cerebrospinal fluid. This method was found to increase the validity and sensitivity of analyses [2]. We derived framewise displacement and percentage of valid volumes (i.e., number of remaining volumes after scrubbing divided by subject's total acquired scan) as covariates in subsequent analyses.

# **Supplementary Tables**

| **Table S1.** Difference in Demographic and Clinical variables between Imaging and Non-Imaging samples (HBN sample) | | | | | | | |  |
| --- | --- | --- | --- | --- | --- | --- | --- | --- |
| Features | Non-Imaging (n=617) |  | Imaging (n=1583) |  | Statistics | | |  |
|  |  |  |  |  | X2/F | p | pFDR |  |
| Age | 10.20 (3.07) |  | 10.86 (3.09) |  | 21.68 | <0.001 | 0.011 |  |
| Sex (Males, %) | 64.20% |  | 62.00% |  | 0.95 | 0.342 | 0.537 |  |
| Recruitment Site | |  |  |  | 7.60 | 0.022 | 0.121 |  |
| Staten Island | 64.70% |  | 61.70% |  |  |  |  |  |
| Mobile Research Vehicule | 2.00% |  | 0.90% |  |  |  |  |  |
| Midtown Manhattan | 33.30% |  | 37.40% |  |  |  |  |  |
| Conduct Problems (CBCL-DSM) | .19 (.4) |  | .2 (.42) |  | 4.38 | 0.617 | 0.766 |  |
| Irritability (ARI-P) | 3.19 (3.31) |  | 3.15 (3.30) |  | 0.08 | 0.781 | 0.797 |  |
| Depression (MFQ) | 8.60 (8.71) |  | 9.26 (9.02) |  | 2.06 | 0.152 | 0.334 |  |
| Anxiety (SCARED) | 14.74 (12.69) |  | 14.45 (11.73) |  | 0.24 | 0.627 | 0.766 |  |
| Internalizing Traits (CBCL) | 9.78 (8.09) |  | 10.36 (8.59) |  | 2.24 | 0.135 | 0.334 |  |
| Callous-Unemotional Traits (ICU) | 24.32 (10.45) |  | 24.17 (10.59) |  | 0.07 | 0.797 | 0.797 |  |
| ADHD symptoms (SWAN-P) | .56 (1.04) |  | .44 (1.04) |  | 1.11 | 0.292 | 0.535 |  |
| Peer Problems (SDQ) | 2.54 (2.22) |  | 2.36 (2.14) |  | 2.98 | 0.084 | 0.308 |  |
| Note. Comparisons on categorical variables were conducted using Chi-square tests. Comparisons on continuous measures were conducted using one-way ANOVAs. Corrections for multiple comparisons were made with Benjamini and Hochberg false discovery rates. | | | | | | | |  |
|  |  |  |  |  |  |  |  |  |

| **Table S2.** Difference in Conduct Problems between Imaging and Non-Imaging samples | | | | | | | | | |  |
| --- | --- | --- | --- | --- | --- | --- | --- | --- | --- | --- |
| Items | Non-Imaging (n=617) | |  | Imaging (n=1583) | |  | Statistics | | |  |
|  | Mean (SD) | % Endorsement (Very True) |  | Mean (SD) | % Endorsement (Very True) |  | F | p | pFDR |  |
| 15. Cruel to Animals | .05 (.23) | 0.30% |  | .03 (.18) | 0.20% |  | 4.469 | 0.035 | 0.310 |  |
| 16. Cruelty, Bullying, Meanness | .19 (.43) | 1.40% |  | .19 (.45) | 2.50% |  | 0.004 | 0.953 | 0.953 |  |
| 21. Destroy Things (Others) | .28 (.53) | 3.80% |  | .26 (.53) | 4.30% |  | 0.877 | 0.349 | 0.539 |  |
| 26. Doesn't seem to feel guilty | .45 (.66) | 9.10% |  | .43 (.66) | 9.30% |  | 0.627 | 0.428 | 0.606 |  |
| 28. Breaks Rules | .64 (.68) | 11.50% |  | .61 (.69) | 12.20% |  | 0.921 | 0.337 | 0.539 |  |
| 37. Gets in Many Fights | .16 (42) | 2.20% |  | .16 (45) | 3.20% |  | 0.067 | 0.796 | 0.949 |  |
| 39. Delinquent Peers | .19 (.47) | 3.00% |  | .24 (.51) | 4.00% |  | 3.648 | 0.056 | 0.310 |  |
| 43. Lying or Cheating | .46 (.62) | 6.60% |  | .51 (.65) | 8.80% |  | 3.799 | 0.051 | 0.310 |  |
| 57. Physically Attacks | .21 (.47) | 2.60% |  | .19 (45) | 2.50% |  | 1.045 | 0.307 | 0.539 |  |
| 67. Runs Away from Home | .02 (.16) | 0.10% |  | .04 (.21) | 0.50% |  | 2.175 | 0.14 | 0.381 |  |
| 72. Sets Fires | .01 (.12) | 1.40% |  | .02 (.14) | 0.20% |  | 0.042 | 0.837 | 0.949 |  |
| 81. Steals at Home | .11 (.37) | 1.80% |  | .14 (.42) | 2.70% |  | 2.435 | 0.119 | 0.381 |  |
| 82. Steals Outside the Home | .06 (.27) | 0.60% |  | .08 (.32) | 1.50% |  | 2.001 | 0.157 | 0.381 |  |
| 90. Swearing | .26 (.54) | 4.80% |  | .26 (.55) | 5.50% |  | 0.005 | 0.946 | 0.953 |  |
| 97. Threatens People | .12 (.38) | 1.80% |  | .13 (.39) | 2.00% |  | 0.433 | 0.51 | 0.667 |  |
| 101. Truancy, Skips School | .05 (.26) | 1.30% |  | .07 (.33) | 2.00% |  | 3.213 | 0.073 | 0.310 |  |
| 106. Vandalism | .03 (.19) | 0.50% |  | .04 (.22) | 0.60% |  | 1.178 | 0.278 | 0.539 |  |
| Note. | | | | | | | | | |  |
|  |  |  |  |  |  |  |  |  |  |  |

| **Table S3.** MRI Scan Parameters of the Healthy Brain Network dataset | | | | | | | |  |
| --- | --- | --- | --- | --- | --- | --- | --- | --- |
|  | Slices | Resolution  (mm) | TR (ms) | TE (ms) | Tl (ms) | Flip angle (deg) | Multi-band |  |
| Staten Island (1.5T Siemens Avanto) | | |  |  |  |  |  |  |
| T1 | 176 | 1.0 x 1.0 x 1.0 | 2730 | 1.64 | 1000 | 7 | Off |  |
| fMRI | 54 | 2.5 x 2.5 x 2.5 | 1450 | 40 | N/A | 55 | 3 |  |
|  |  |  |  |  |  |  |  |  |
| RUBIC (3T Siemens Trio Tim) | | |  |  |  |  |  |  |
| T1 | 224 | 0.8 x 0.8 x 0.8 | 2500 | 3.15 | 1060 | 8 | Off |  |
| fMRI | 60 | 2.4 x 2.4 x 2.4 | 800 | 30 | N/A | 31 | 6 |  |
|  |  |  |  |  |  |  |  |  |
| CBIC (3T Siemens Prisma) | | |  |  |  |  |  |  |
| T1 | 224 | 0.8 x 0.8 x 0.8 | 2500 | 3.15 | 1060 | 8 | Off |  |
| fMRI | 60 | 2.4 x 2.4 x 2.4 | 800 | 30 | N/A | 31 | 6 |  |
| *Note.* RUBIC = Rutgers University Brain Imaging Center; CBIC = CitiGroup Cornell Brain Imaging Center | | | | | | | |  |
|  |  |  |  |  |  |  |  |  |

| **Table S4.** Information about the Autism Brain Imaging Data Exchange datasets of Healthy Subjects (n=598) | | | | | | | |
| --- | --- | --- | --- | --- | --- | --- | --- |
| Datasets | N | Age | Boys | Manufacturer | Model | Tesla | TR |
| **ABIDE-I** |  |  |  |  |  |  |  |
| University of Pittsburgh (PITT) | 9 | 14.28 (1.62) | 88.89 | Siemens | Allegra | 3.0 | 1.50 |
| Olin Neuropsychiatry Research Center (OLIN) | 3 | 15.67 (1.154) | 66.67 | Siemens | Allegra | 3.0 | 1.50 |
| Oregon Health and Science University (OHSU) | 11 | 9.89 (1.03) | 100.00 | Siemens | TriTim | 3.0 | 2.50 |
| San Diego State University (SDSU) | 21 | 14.17 (1.93) | 71.43 | GE | MR750 | 3.0 | 2.00 |
| Trinity Centre for Health Sciences (TRINITY) | 12 | 14.82 (1.67) | 100.00 | Philips | Achieva | 3.0 | 2.01 |
| University of Michigan (UM_1) | 39 | 13.58 (2.87) | 71.79 | GE | SIgna | 3.0 | 2.00 |
| University of Michigan (UM_2) | 17 | 15.66 (1.58) | 94.12 | GE | SIgna | 3.0 | 2.00 |
| University of Utah School of Medicine (USM) | 10 | 14.23 (2.47) | 100.00 | SIemens | Tritim | 3.0 | 2.00 |
| Yale Child Study Center (YALE) | 23 | 12.75 (2.95) | 69.57 | Siemens | TriTim | 3.0 | 2.00 |
| University of Leuven (LEUVEN_2) | 16 | 14.56 (1.57) | 75.00 | Philips | Intera | 3.0 | 1.68 |
| Kennedy Krieger Institute (KKI) | 24 | 10.18 (1.35) | 66.67 | Philips | Achieva | 3.0 | 2.50 |
| NYU Langone Medical Center (NYU) | 63 | 12.34 (3.07) | 73.02 | Siemens | Allegra | 3.0 | 2.00 |
| Standord University (STANFORD) | 14 | 10.07 (1.42) | 78.57 | GE | Signa | 3.0 | 2.00 |
| University of California, Los Angeles (UCLA_1) | 24 | 13.98 (1.81) | 83.33 | Siemens | TriTim | 3.0 | 3.00 |
| University of California, Los Angeles (UCLA_2) | 9 | 12.58 (0.944) | 70.00 | Siemens | TriTim | 3.0 | 3.00 |
| Ludwig Maximilians University Munich (MAXMUN) | 4 | 11.0 (3.74) | 100.00 | Siemens | Verio | 3.0 | 3.00 |
| California Institute of Technology (CALTECH) | 1 | 17 (-) | 100.00 | Siemens | TriTim | 3.0 | 2.00 |
|  |  |  |  |  |  |  |  |
| **ABIDE II** |  |  |  |  |  |  |  |
| Georgetown University (GU) | 40 | 10.77 (1.70) | 52.50 | SIemens | Tritim | 3.0 | 1.50 |
| San Diego State University (SDSU) | 22 | 13.06 (3.04) | 90.91 | GE | MR750 | 3.0 | 2.00 |
| Oregon Health and Science University (OHSU) | 49 | 10.47 (1.68) | 46.94 | Siemens | Tritim | 3.0 | 2.50 |
| Trinity Centre for Health Sciences (TCD) | 8 | 14.43 (1.20) | 100.00 | Philips | Achieva | 3.0 | 2.00 |
| NYU Langone Medical Center (NYU) | 23 | 9.32 (1.90) | 100.00 | Siemens | Allegra | 3.0 | 2.00 |
| Kennedy Krieger Institute (KKI) | 109 | 10.31 (1.19) | 59.63 | Philips | Achieva | 3.0 | 2.50 |
| University of Utah School of Medicine (USM) | 2 | 12.67 (1.24) | 100.00 | SIemens | Tritim | 3.0 | 2.00 |
| Institute Pasteur & Robert Debré Hospital (IP) | 5 | 12.13 (3.10) | 0.00 | Philips | Achieva | 1.5 | 2.70 |
| University of California, Los Angeles (UCLA) | 12 | 10.20 (2.26) | 75.00 | SIemens | Tritim | 3.0 | 3.00 |
| Erasmus University Medical Center (EMC) | 16 | 8.01 (.91) | 75.00 | GE | MR750 | 3.0 | 2.00 |
| University of California Davis (UCD) | 12 | 15.23 (1.44) | 66.67 | Siemens | TriTim | 3.0 | 2.00 |
| Note. | | | | | | | |

| **Table S5**. Results of the exploratory factor analysis (pattern matrix) | | |  |
| --- | --- | --- | --- |
| Items (DSM-Oriented Conduct Problems) | Factors | |  |
|  | **Factor 1**  Aggression | **Factor 2**  Rule-Breaking |  |
| 15. Cruel to animals | 0.401 | - |  |
| 16. Cruelty, bullying, or meanness to others | 0.806 | - |  |
| 37. Gets in many fights | 0.714 | - |  |
| 57. Physically attacks people | 0.789 | - |  |
| 97. Threatens people | 0.672 | - |  |
| 21. Destroys things belonging to his/her family or others | - | - |  |
| 26. Doesn't seem to feel guilty after misbehaving | - | - |  |
| 28. Breaks rules at home, school, or elsewhere | - | 0.461 |  |
| 39. Hangs around with others who get in trouble | - | - |  |
| 43. Lying or cheating | - | 0.655 |  |
| 67. Runs away from home | - | - |  |
| 72. Sets fires | - | - |  |
| 81. Steals at home | - | 0.751 |  |
| 82. Steals outside the home | - | 0.610 |  |
| 90. Swearing or obscene language | - | - |  |
| 101. Truancy, skips school | - | - |  |
| 106. Vandalism | - | - |  |
| *Note.* Promax rotation. Items with coefficients with less than .40 were removed. | | |  |
|  |  |  |  |

| **Table S6.** Analyses on fALFF correlates after considering Internalizing Traits as a potential confounder | | | | | | |
| --- | --- | --- | --- | --- | --- | --- |
| Brain Regions (Composite Score) | Direction (+/-) | MNI Coordinates | | | t-value | cluster size |
|  |  | x | y | z |  |  |
| **Conduct Problems** |  |  |  |  |  |  |
| Postcentral Gyrus | + | -60 | -20 | 28 | 4.19 | 40 |
| **Precentral Gyrus** | **+** | **38** | **-24** | **64** | **3.99** | **71** |
| Superior Frontal Gyrus | + | 12 | 26 | 62 | 3.87 | 27 |
| Subgenual Anterior Cingulate Cortex | - | 12 | 24 | -12 | 4.41 | 21 |
| **Temporo-Parietal Junction (Inferior Parietal Lobule)** | **-** | **52** | **-46** | **22** | **4.22** | **59** |
| Inferior Temporal Gyrus | - | -52 | -50 | -26 | 4.04 | 37 |
| Inferior Frontal Gyrus | - | 40 | 12 | 22 | 3.9 | 28 |
| **Superior Temporal Gyrus (posterior)** | **-** | **56** | **-26** | **6** | **3.82** | **36** |
| Superior Temporal Gyrus (anterior) | - | -56 | -4 | -10 | 3.69 | 32 |
|  |  |  |  |  |  |  |
| **Aggression** |  |  |  |  |  |  |
| **Primary Motor Cortex** | **+** | **-10** | **-14** | **74** | **4.58** | **41** |
| Precentral Gyrus | + | 40 | -22 | 68 | 3.89 | 45 |
| Lateral Occipital Cortex | + | -40 | -88 | -8 | 3.88 | 46 |
| Postcentral Gyrus | + | -62 | -20 | 26 | 3.85 | 20 |
| **Posterior Parahippocampal Gyrus** | **-** | **32** | **-40** | **-18** | **4.15** | **23** |
| **Mid Superior Temporal Gyrus** | **-** | **58** | **-12** | **4** | **4.13** | **25** |
| **Temporo-Parietal Junction (Inferior Parietal Lobule)** | **-** | **52** | **-40** | **22** | **3.86** | **36** |
|  |  |  |  |  |  |  |
| **Rule-Breaking** |  |  |  |  |  |  |
| **Precentral Gyrus** | **+** | **40** | **-24** | **64** | **4.39** | **147** |
| Primary Motor Cortex | + | -10 | -12 | 74 | 4.27 | 54 |
| Postcentral Gyrus | + | -62 | -20 | 26 | 4.05 | 29 |
| Lateral Occipital Cortex | + | -42 | -88 | -6 | 3.83 | 44 |
| Superior Frontal Gyrus | + | -20 | 22 | 60 | 3.7 | 21 |
| Middle Temporal Gyrus (anterior) | - | -62 | -2 | -12 | 4.3 | 35 |
| Lobule VI | - | 16 | -66 | -22 | 4.18 | 27 |
| Calcarine Cortex | - | 0 | -64 | 12 | 4.05 | 21 |
| **Superior Temporal Gyrus (posterior)** | **-** | **58** | **-28** | **6** | **4.03** | **21** |
| Middle Temporal Gyrus | - | -44 | -58 | 4 | 3.96 | 28 |
| **Precuneus** | **-** | **2** | **-70** | **28** | **3.82** | **53** |
| **Middle Temporal Gyrus** | **-** | **-58** | **-30** | **-2** | **3.57** | **23** |
| Note. **BOLD** represents results that were reported in the initial analyses without internalizing traits as a potential confounder. | | | | | | |

| **Table S7.** Findings from the Amplitude Low Frequency Fluctuations (ALFF) analyses. | | | | | | |
| --- | --- | --- | --- | --- | --- | --- |
| Brain Regions | Direction (+/-) | MNI Coordinates | | | t-value | cluster size |
|  |  | x | y | z |  |  |
| **SHARED EFFECT** (CP ∩ AGG ∩ RB) |  |  |  |  |  |  |
| **Frontal Pole** |  |  |  |  |  |  |
| Conduct Problems | + | -30 | 66 | -4 | 4.21 | 183* |
| Aggression | + | -36 | 62 | 2 | 4.00 | 111 |
| Rule-Breaking | + | -30 | 64 | -4 | 3.79 | 26 |
| **Precentral Gyrus** |  |  |  |  |  |  |
| Conduct Problems | + | 38 | -22 | 68 | 4.05 | 37 |
| Aggression | + | 38 | -22 | 68 | 4.01 | 31 |
| Rule-Breaking | + | 40 | -24 | 68 | 4.02 | 51 |
| **Temporo-Parietal Junction (Inferior Parietal Lobule)** | |  |  |  |  |  |
| Conduct Problems | - | 48 | -50 | 24 | 4.93 | 235* |
| Aggression | - | 54 | -44 | 14 | 3.85 | 76 |
| Rule-Breaking | - | 54 | -46 | 22 | 4.14 | 88 |
| **Anterior Cingulate Cortex (perigenual, subgenual)** | |  |  |  |  |  |
| Conduct Problems | - | 8 | 36 | -4 | 4.25 | 92 |
| Aggression | - | 8 | 36 | -4 | 4.38 | 64 |
| Rule-Breaking | - | 10 | 22 | -12 | 4.03 | 55 |
| **ventrolateral PFC/lateral OFC** |  |  |  |  |  |  |
| Conduct Problems | - | 46 | 36 | -10 | 4.13 | 29 |
| Aggression | - | 46 | 38 | -10 | 3.88 | 26 |
| Rule-Breaking | - | 40 | 32 | -12 | 4.34 | 102 |
| **Planum Temporale** |  |  |  |  |  |  |
| Conduct Problems | - | 58 | -10 | 4 | 3.87 | 26 |
| Aggression | - | 60 | -10 | 4 | 4.40 | 48 |
| Rule-Breaking | - | 58 | -12 | 4 | 4.15 | 38 |
| **Superior Temporal Gyrus (posterior)** |  |  |  |  |  |  |
| Conduct Problems | - | 54 | -28 | 8 | 3.78 | 44 |
| Aggression | - | 56 | -26 | 8 | 3.92 | 32 |
| Rule-Breaking | - | 58 | -30 | 8 | 3.77 | 25 |
| **UNIDIMENSIONAL LEVEL EFFECT** (CP-specific) | |  |  |  |  |  |
| **pre-Supplementary Motor Area** |  |  |  |  |  |  |
| Conduct Problems | + | 14 | 30 | 62 | 3.86 | 22 |
| **Superior Frontal Gyrus** |  |  |  |  |  |  |
| Conduct Problems | - | 22 | 30 | 42 | 3.88 | 25 |
| **Inferior Temporal Cortex (posterior)** |  |  |  |  |  |  |
| Conduct Problems | - | 56 | -48 | -12 | 3.79 | 20 |
| **AGGRESSION EFFECT** (AGG ∩ CP; AGG-specific) | | |  |  |  |  |
| **Frontal Pole** |  |  |  |  |  |  |
| Conduct Problems | + | 38 | 60 | -16 | 3.88 | 73 |
| Aggression | + | 30 | 66 | -10 | 3.77 | 56 |
| **dorsomedial PFC** |  |  |  |  |  |  |
| Conduct Problems | - | 10 | 46 | 36 | 3.94 | 36 |
| Aggression | - | 10 | 46 | 36 | 3.61 | 36 |
| **Secondary Somatosensory** |  |  |  |  |  |  |
| Conduct Problems | - | 44 | -34 | 28 | 3.74 | 20 |
| Aggression | - | 46 | -32 | 28 | 3.98 | 24 |
| **medial PFC** |  |  |  |  |  |  |
| Aggression | - | 6 | 46 | 14 | 4.27 | 22 |
| **RULE-BREAKING EFFECT** (RB ∩ CP; RB-specific) | | |  |  |  |  |
| **Visual Area (V4)** |  |  |  |  |  |  |
| Rule-Breaking | - | -42 | -92 | -4 | 4.16 | 52 |
| **Lobule VI** |  |  |  |  |  |  |
| Rule-Breaking | - | -20 | -64 | -22 | 4.25 | 26 |
| **Lobule VI** |  |  |  |  |  |  |
| Rule-Breaking | - | 18 | -68 | -22 | 4.00 | 24 |
| **Lobule VIIb** |  |  |  |  |  |  |
| Rule-Breaking | - | 18 | -74 | -44 | 3.60 | 24 |
| **FINE-GRAINED LEVEL EFFECT** (shared AGG ∩ RB, but not CP) | | |  |  |  |  |
| **Superior Temporal Gyrus (anterior)** |  |  |  |  |  |  |
| Aggression | - | 60 | -6 | -8 | 3.76 | 22 |
| Rule-Breaking | - | 60 | -4 | -8 | 3.84 | 38 |
| **Note.** Findings are thresholded at p<0.001, 20 voxels. * = survived pFWE<0.05 at a cluster-level. Models are adjusted for effect of age, sex, sites, percentage of valid scans and mean motion. MNI =Montreal Neurological Institute; PFC = Prefrontal Cortex; OFC = Orbitofrontal Cortex | | | | | | |
|  |  |  |  |  |  |  |

| **Table S8.** Effect sizes of the importance of intrinsic functional conectivity networks | | | | |
| --- | --- | --- | --- | --- |
| Networks | Conduct Problems | Aggression | Rule-Breaking | Aggression > Rule-Breaking |
| Fronto-Parietal | -0.524 | -0.700 | -0.655 | -0.038 |
| Dorsal Attention | -0.197 | 0.041 | -0.709 | 0.690 |
| Default Mode | 0.396 | -0.267 | 1.627 | -1.814 |
| Limbic | -0.237 | -0.273 | 0.040 | -0.288 |
| Subcortex | 0.156 | 0.512 | 0.051 | 0.423 |
| Somatomotor | 2.721 | 2.745 | 1.075 | 1.182 |
| Visual | -0.056 | 0.595 | 0.747 | -0.134 |
| Ventral Attention | 1.221 | 1.244 | -0.392 | 1.541 |
| Note. | | | | |
|  |  |  |  |  |

| **Table S9.** Spatial Associations between Functional Connectivity Maps and Mental Functions | | | | | | | |  |
| --- | --- | --- | --- | --- | --- | --- | --- | --- |
| Mental functions | Conduct Problems | | |  | Aggression | | |  |
|  | r-to-z | p-value | pFDR |  | r-to-z | p-value | pFDR |  |
| Action | 0.127 | 0.0144 | 0.0312 |  | 0.286 | 0.0002 | 0.0009 |  |
| Value-Based Decision-Making | -0.192 | 0.2188 | 0.3160 |  | -0.350 | 0.0300 | 0.0487 |  |
| Motivation | -0.093 | 0.4959 | 0.5861 |  | -0.089 | 0.5163 | 0.6102 |  |
| Social Representation | 0.030 | 0.8314 | 0.8396 |  | -0.234 | 0.0960 | 0.1248 |  |
| Multiple-Demand | -0.067 | 0.1746 | 0.2837 |  | -0.112 | 0.0208 | 0.0386 |  |
| Face Detection | 0.036 | 0.4659 | 0.5861 |  | 0.117 | 0.0176 | 0.0381 |  |
| Language | 0.079 | 0.1058 | 0.1964 |  | -0.021 | 0.6639 | 0.6639 |  |
| Spatial Memory | -0.197 | 0.0002 | 0.0006 |  | 0.028 | 0.5675 | 0.6148 |  |
| Cognitive Control | -0.259 | 0.0002 | 0.0006 |  | -0.185 | 0.0004 | 0.0013 |  |
| Physiological Arousal | 0.392 | 0.0002 | 0.0006 |  | 0.349 | 0.0002 | 0.0009 |  |
| Auditory Perception | 0.573 | 0.0002 | 0.0006 |  | 0.511 | 0.0002 | 0.0009 |  |
| Social Inference | -0.024 | 0.8396 | 0.8396 |  | -0.251 | 0.0652 | 0.0942 |  |
| Spatial Attention | -0.380 | 0.0038 | 0.0099 |  | -0.358 | 0.0034 | 0.0088 |  |
|  |  |  |  |  |  |  |  |  |
| Mental functions | Rule-Breaking | | |  | Aggression > Rule-Breaking | | |  |
|  | r-to-z | p-value | pFDR |  | r-to-z | p-value | pFDR |  |
| Action | -0.424 | 0.000 | 0.001 |  | 0.618 | 0.0002 | 0.0004 |  |
| Value-Based Decision-Making | 0.050 | 0.680 | 0.773 |  | -0.318 | 0.0410 | 0.0484 |  |
| Motivation | -0.182 | 0.105 | 0.171 |  | 0.085 | 0.5031 | 0.5031 |  |
| Social Representation | 0.111 | 0.322 | 0.465 |  | -0.294 | 0.0308 | 0.0445 |  |
| Multiple-Demand | -0.579 | 0.000 | 0.001 |  | 0.308 | 0.0002 | 0.0004 |  |
| Face Detection | -0.373 | 0.000 | 0.001 |  | 0.306 | 0.0002 | 0.0004 |  |
| Language | -0.145 | 0.003 | 0.005 |  | 0.124 | 0.0104 | 0.0169 |  |
| Spatial Memory | 0.041 | 0.399 | 0.518 |  | -0.055 | 0.2656 | 0.2877 |  |
| Cognitive Control | -0.670 | 0.000 | 0.001 |  | 0.268 | 0.0002 | 0.0004 |  |
| Physiological Arousal | -0.226 | 0.000 | 0.001 |  | 0.600 | 0.0002 | 0.0004 |  |
| Auditory Perception | -0.014 | 0.766 | 0.773 |  | 0.487 | 0.0002 | 0.0004 |  |
| Social Inference | 0.395 | 0.001 | 0.002 |  | -0.555 | 0.0002 | 0.0004 |  |
| Spatial Attention | -0.028 | 0.773 | 0.773 |  | -0.244 | 0.0392 | 0.0484 |  |
| Note. | | | | | | | |  |
|  |  |  |  |  |  |  |  |  |

# **Supplementary Figures**

**
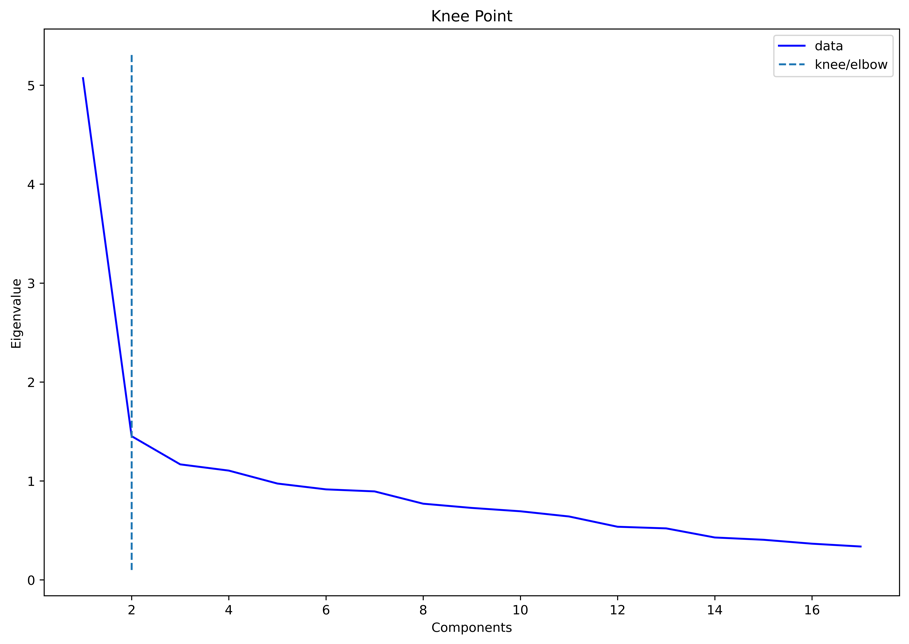
**

## **Figure S1.** Scree plot revealed a maximum curvature at 2 Factors

**Figure S2.** Findings from the Amplitude Low Frequency Fluctuations (ALFF) analyses on **A.** Conduct Problems, **B.** Spatial Overlap in brain regions positively correlated with AGG and RB, and **C.** negatively with AGG and RB. Findings were adjusted for the effect of age, sex, sites, percentage of valid scans and mean motion, and thresholded using p<0.001 uncorrected with 20 voxels.

**Figure S3.** Normative Functional Connectivity Map derived from the regional deficits fALFF for Conduct Problems. Ridge plots represent the distribution of correlation between functional connectivity map and mental functions (Dugré et Potvin, 2023) for each of the 598 healthy adolescents. Bar Graphs represent the effect size (Cohen's d) of the importance of intrinsic connectivity networks in the CP map.

# **Supplementary Results**

Amplitude Low Frequency Fluctuation (ALFF)

*Unidimensional effect: Conduct Problems*

Composite score (Unidimensional CP) was positively associated with ALFF of the bilateral frontal poles (FP), right precentral gyrus (PreCG) and right pre-supplementary motor area (pre-SMA). Findings also revealed negative association with various frontal (perigenual- subgenual anterior cingulate cortex, dorsomedial and lateral PFC) and posterior temporal regions (temporo-parietal junction/posterior superior temporal sulcus (TPJ/pSTS), posterior superior and inferior temporal gyri) (Supplementary Table 2, Supplementary Figure 2). The left FP and right TPJ/pSTS clusters survived FWE correction (pFWE<0.05)

*Shared and Specific Effects of Behavioral Phenotypes*

Composite score of RB replicated the CP-related findings in the left FP, the right PreCG, TPJ, ACC, vlPFC, Planum Temporale, and posterior STG. It also revealed additional RB-specific effect in the visual area (V4) and cerebellar regions (VI, VIIb) (Table S7, Figure S2).

Using AGG-Composite score revealed similar CP-related findings in the left FP, the right PreCG, TPJ, ACC, vlPFC, Planum Temporale, posterior STG, but also the right FP, dmPFC and SII. It also revealed a specific effect in the medial PFC (Table S7, Figure S2).

**REFERENCES**

1. Behzadi Y, Restom K, Liau J, Liu TT (2007) A component based noise correction method (CompCor) for BOLD and perfusion based fMRI. Neuroimage 37:90-101

2. Chai XJ, Castañón AN, Ongür D, Whitfield-Gabrieli S (2012) Anticorrelations in resting state networks without global signal regression. Neuroimage 59:1420-1428

3. Power JD, Mitra A, Laumann TO, Snyder AZ, Schlaggar BL, Petersen SEJN (2014) Methods to detect, characterize, and remove motion artifact in resting state fMRI. 84:320-341

4. Whitfield-Gabrieli S, Nieto-Castanon A (2012) Conn: a functional connectivity toolbox for correlated and anticorrelated brain networks. Brain Connect 2:125-141
